# Volume X

**Published:** 1889-01

**Authors:** 


### Editorial.

### VOLUME X.

The present time seems to mark an era in the history of the onward movement of Dentistry. The spirit of organization seems to have taken hold of the entire body. In our estimation, no one step has been of more importance than the organization of the International Dental Publication Company. The times seem to have been ripe for such a movement and even its most sanguine promulgators have been almost startled at its success from its very incipiency. The profession is to be congratulated that, at last, a strong healthy organ, sustained by over one hundred of the best men in its ranks, has been established. Having no interests but that of the profession at large, its criticisms shall be outspoken and its judgment impartial. If an abuse needs correcting its columns shall always be open for as full ventilation as the occasion demands. Such has been the tone of the journal for years, and a glance at its advertising columns is all that is needed to convince any person that its independence is fully appreciated, and that the manufacturing interests have full confidence in the ultimate success of the Journal, and are willing to help establish it. Had we had the time, there is no doubt that we could have materially increased our advertising. What we have has come to us voluntarily, or through correspondence. No more substantial proof could be adduced that there was a need for just such a movement.

We also feel proud of our subscription list which is rapidly increasing; our readers numbering at the present time nearly two thousand five hundred of the most progressive men in the profession. Our foreign circulation equals or exceeds that of any other American journal published. Our list of foreign correspondents, although not as yet complete, numbers some of the very brightest men abroad.

It has been said that the movement was evanescent, and would soon die out. That is farthest from the truth. It is gaining strength every day. Its influence is far-reaching, and will in time penetrate every portion of the country where English is spoken and dentistry practised.

It has also been queried as to what was independent journalism; and, in reply we will say, journalism that will stand for the good of the entire profession in everything that pertains to the general good regardless of self-interest. A journalism that will grasp at any progressive movement, and fully and heartily second it without first having to "consult its attorney." A journalism that is in harmony with the profession at large and needs no apology for its position. A journalism that knows no interest except that of the dental profession for which it is published, because it is owned and controlled by members of that body. A journalism in which journalism is first, and trade a secondary matter. A journalism that tries to inculcate a professional spirit, and stir up a personal pride in all that relates to dentistry, and, lastly, a journalism that will in time receive the full support of every man who loves his profession and desires to see its advancement.

In order that the entire body of practising dentists may have an opportunity of knowing of the aims and purposes of the INTERNATIONAL DENTAL JOURNAL, we appeal to each and every subscriber to present its claims to the brother practitioners with whom he comes in contact. We appeal to you for financial support for the only journal that can make any just claim to being the organ of the profession.

